# The Dark Side of Social Media: Content Effects on the Relationship Between Materialism and Consumption Behaviors

**DOI:** 10.3389/fpsyg.2022.870614

**Published:** 2022-04-28

**Authors:** Alfonso Pellegrino, Masato Abe, Randall Shannon

**Affiliations:** ^1^College of Management, Mahidol University, Bangkok, Thailand; ^2^UNESCAP, Bangkok, Thailand

**Keywords:** materialism and consumerism, negative consumption, impulse buying behavior, social media marketing strategy, conspicuous consumption

## Abstract

This study contributes to the emerging literature on the negative effects over consumption that social media users may develop as a consequence of being engaged on social media platforms. The authors tested materialism’s direct and indirect impacts on compulsive, conspicuous, and impulsive buying, adding two novel mediators: attitudes toward social media content (SCM) and social media intensity (SMI). The study uses a convenience sample of 400 Thai social media users analyzed using structural equation modeling. The results confirmed the well-established positive relationships between materialism and each of the three-negative consumption behaviors also in the social media domain. A novel finding showed the important role played by SMI which was found to be a strong predictor of each of the three negative consumption behaviors and it was also found to significantly mediate the relationship between materialism and the three-negative consumption behaviors. An additional contribution of the study was found on the role of attitudes which, contrary to what is commonly believed, were often not significant in predicting any negative behavior.

## Introduction

The study objective is shading light on new and never tested relationships on factors that affect social media users developing negative consumption behaviors as a consequence of being engaged on social media platforms. The study contributes to the literature by offering several innovative position regarding human media consumption interaction. Firstly, the authors adopted the notion that sees materialism as a personality trait and therefore the construct is treated as an antecedent of attitudes and social media intensity (SMI) differently by what has been stated by traditional literature which see materialism as a consequence of prolonged exposure to advertisement messages. Secondly, the study tested the well-established relationships between materialism and compulsive, conspicuous, and impulsive buying behavior within the social media domain and lastly two new mediators namely attitudes toward social media content (SCM) and SMI, were tested in order to check which one could better explain the relationship between materialism and each of the negative consumption behaviors.

Testing and examining the three different negative consumption behaviors is justified in this study as we want to gain a comprehensive overview of materialism, attitudes and SMI’s impact over different types of consumption behaviors. Impulse buying is more associated with external triggers (Wang, 2015) that stimulate an individual’s impulsive nature, resulting in an impulse to buy. Ads customization, promotion, sales, and shopping environment all contribute to an increase an individual’s impulsive nature, leading to an urge to buy. This is often the case for online consumers with high SMI constantly browsing social media pages who are often exposed to promotion and heavily customized advertising materials, compulsive buying behavior instead has been linked to potential causes that are “biochemical, psychological, or societal in nature” (Faber and Christenson, 1996, p. 804). Compulsive buying behavior is characterized by “uncontrolled and excessive purchasing” (Billieux et al., 2008, p. 1432) and “excessive or poorly controlled preoccupations, urges, or behaviors regarding spending” (Black, 2001, p. 17) triggered by internal tension, often involving frustration that can “only be relieved by purchases” (Billieux et al., 2008, p. 1433). Consumers may obtain a short-term and transient benefit after being rewarded for their actions, a surge of pleasant effects provide compulsive purchasers with a means of modifying and improving their mood, at least for the time being. Conspicuous buying behavior was included next to compulsive and impulse buying behavior, as it has been shown to be often related with social media users, who often consume following herd behavior to gain social proof from their peers and constantly compare themselves with influencers and their peers. Impulsive buyers appear to be buying products also conspicuously as the tend to buy flashy products they had previously seen from influencers or other users deemed experts in their product category.

The rationale for carrying out the study is that we are now entering into stage 4 of online relationship marketing, where the communication between brands and users is no longer one-way, but rather interactive (Thaichon et al., 2020). New technology changes the nature of how advertising and interactions work, which consequently impacts how companies need to convey their message to customers. More aggressive and tailored messages launched on social media platforms may enhance negative consumption behaviors and there can be dire consequences for countries’ economies and for individuals’ indulging in these behaviors; economically users are more likely to incur into higher debts, credit card over usage which can lead to family problems, mental disorders, addiction (Buja et al., 2018), depression (Yoon et al., 2019), and low self-esteem (Dumas et al., 2020). on a macro scale having too many citizens who are unable to pay for their debt may even trigger financial crisis; environmentally production of resource intensive products which can lead to pollution and natural resources depletion and socially labor-intensive production leading to labor exploitation. These are just some of the most awful consequences of engaging in any of these behaviors. The study sample has been collected from social media pages of well-known e-commerce platforms, which frequently use heavily customized paid ads, also known as retargeting ads, whose negative effects on consumption and on the mental well-being of social media users have been reported in previous literature (Lulandala, 2020). Thailand is among the most social media engaged population in the world having 51 million users on Facebook and 16 million users on Instagram, however, very little focus has been placed on investigating the characteristics of compulsive, impulsive and conspicuous consumption as effects of social media usage (Gupta and Vohra, 2019).

According to Changchit et al. (2019), Thais’ social media usage increased between 2015 and 2018 from 18 to 32 h per week, while their online spending almost doubled during this period.

Seventy percent of social media users in one survey reported that they were likely to click on advertising in their newsfeed (Digital, 2021). According to Digital (2021), a social media marketing agency, Facebook (38%) and Instagram (37%) are the platforms with the highest percentages of advertisements generating clicks. Digital (2021) reported that there were 55 million internet users in Thailand in January 2021, while the number of social media users in the country increased by 2.3 million (4.7%) between April 2020 and January 2021 Digital (2021) also revealed that 78% of the Thai population are active social media users, ranking Thailand fourth globally for social networking engagement hence the focus on SMI variable. Therefore, the first objective of this research is to determine whether materialism directly enhances the three negative behaviors also in the social media domain. Secondly, we aim to identify the extent to which the relationships between materialism and the three-negative consumption behaviors are mediated by attitudes toward SCM and by SMI.

## Literature Review

### Materialism

Inglehart (1981) claimed that materialists place a higher value on worldly attainment over spiritual attainment. Along with his definition, he developed the theory of post-materialism. Belk (1985) conceived materialism as the manifestation of three personality characteristics: greediness, non-generosity and envy. By quantifying these three features, human materialism can be measured. Possessiveness is defined as the desire to own or control one’s own property. Non-generosity is viewed as a reluctance to share one’s own belongings with others. Jealousy is defined as a desire for someone else’s wealth (Belk, 1982). Another definition of materialism is called the personal values, which was developed by Richins and Dawson (1992). They argued that materialism is a system of personal values. They used three concepts of materialism: centrality, happiness, and success. Centrality is a prominent focus on physical objects in a person’s life. According to Tooby and Cosmides (1992) human organisms are inherently empty and materialism acquires meaning in accord with social and cultural teachings, this view is consistent with opinions expressed by Larsen et al. (1999) which see materialism as an individual phenomenon, that label a person who values material objects highly. Larsen et al. (1999) Larsen noted that materialism is not innate, but acquired through cultural teachings. He noted that religious perspective holds that human beings are born corrupt or fallen, with an unholy and unreasonable desire to amass things. According to Sirgy et al. (2012) materialism may be both good and bad. He developed a model that reconciled these two contrasting viewpoints by asserting that materialism may lead to life dissatisfaction when materialistic people evaluate their standard of living using fantasy-based expectations (e.g., ideal expectations), which increases the likelihood that they would evaluate their standard of living negatively. In turn, dissatisfaction with standard of living increases the likelihood that they would evaluate their life negatively. However, materialistic people who evaluate their standard of living using reality-based expectations are likely to feel more economically motivated than their non-materialistic counterparts, and this economic motivation is likely to contribute significantly and positively to life satisfaction. Shrum et al. (2013) stresses the functions of materialistic goal pursuit, the processes by which these functions are developed and implemented, and their potential consequences. This functional perspective views materialistic behavior as motivated goal pursuit intended to construct and maintain self-identity, and defines materialism as the extent to which people engage in identity maintenance and construction through symbolic consumption.

The literature on materialism in relation to social media and online consumption behavior reveals that social networking sites provide outlets for customers to access, distribute and exchange information of different kinds regarding products and experiences. Given the increasing popularity of these sites, marketers employ them to communicate with their potential consumers. Bush and Gilbert (2002) found that consumers who spend more time on social media have higher levels of materialism than those who spend time reading newspapers. Likewise, Kamal et al. (2013) recently looked at this relationship and discovered that customers appear to have higher rates of materialism when their social network intensity of usage increases. While studying more recent research, it is necessary to understand that social media users build an identity that portrays themselves in order to encourage engagement from other users (Haferkamp and Krämer, 2011).

Unlike television, social media usage occurs mainly individually and in more private environments. Social networking discussions can be as genuine as people’s conversations (Mangold and Faulds, 2009). Therefore, the impact of advertising content on social media is more personal than on television (Fournier and Avery, 2011). With customers always having access to social media through smartphones, in different contextual settings of time, place, and even state of mind, messages from brands or users can reach users anytime. Television programming prepares the audience for when they will see the ads by showing them between shows, whereas social media advertising messages have various degrees of ad placements impacting the users more profoundly depending on their emotional state of mind (Joyce and Harwood, 2014). Thus, social media marketing practices target the individual users directly, in an intimate and personal setting, and have potentially higher levels of impact on creating materialistic desires, resulting in subsequent increase in consumption patterns in the market.

### Attitudes Toward Social Media Content

People’s attitudes are produced spontaneously and consistently from their beliefs accessible in their memory, which then guide their corresponding behaviors. Attitudes were defined by Armstrong and Kotler (2000) as people’s emotional responses to any paid form of non-personal presentation and promotion by an identified sponsor of ideas, products or services. Park and Lin (2018) found that over 20% of the consumers in their study believed that the attitudes they had toward SCM were important to their final purchase decision, while another 20% stated that their attitudes helped them decide what to purchase. Social media research reveals that positive attitudes and higher levels of intention to buy are associated with success tales and high popularity, as opposed to failure stories and low popularity. The detrimental impact of failure stories on users’ attitudes is mitigated by their popularity (Pahlevan Sharif and Mura, 2019).

### Social Media Intensity

Social media intensity is defined as a social media user’s level of activity and engagement with social media (Ellison et al., 2007). It refers to the strength of involvement in the activity itself and the extent to which social media platforms are integrated into people’s everyday lives.

However, when reviewing empirical studies on the link between SMI and compulsive buying, it was discovered that the literature on the subject is limited. Trotzke et al. (2015) attempted to establish a link between SMI and compulsive buying behavior by claiming that the former provided an outlet for those who already have a tendency to seek escape in the form of shopping to alleviate negative situations or circumstances, implying that SMI does have an impact. Based on previous research on the impact of social networking on self-image and self-control (Khan and Dhar, 2006; Wilcox et al., 2011), found that the frequency with which people use social media causes them to make irrational decisions by increasing their spending on luxurious goods, also known as conspicuous goods. Previous research has found that the factors which influence online impulse purchases include photographs of products, banner ads, low prices and exclusive deals (de Kervenoael et al., 2009). The question of whether personalized content influence online purchases is based on previous research which established an intimate link between the self and impulse purchases (Dittmar and Drury, 2000).

### Compulsive Shopping Behavior in Relation to Social Media

Compulsive shopping behavior was defined by O’Guinn and Faber (1989) as a chronic habit of purchasing goods repeatedly and excessively. It is characterized by excessive or poorly controlled preoccupations, urges or behaviors regarding shopping and spending, which then lead to adverse consequences. Research in the social media domain has shown that compulsive buying is significantly affected by intense platform usage (Kukar-Kinney et al., 2009; Sharif and Khanekharab, 2017). Following the social comparison theory (Festinger, 1954), social media sites are where users can examine and compare aspects of their lives with those of their peers. Similarly, social identity formation is greatly impacted by the individual’s association with social media influencers and/or membership of desirable communities (Tajfel, 1981). Research show that social comparison is a key mechanism impacting social media users’ subjective wellbeing and users with a tendency to engage in social comparison are especially likely to be negatively impacted by SCM (Tiggemann et al., 2018; Verduyn et al., 2020; Zheng et al., 2020).

Many consumers prefer shopping on social media platforms because of its convenience and ease of use, as it allows them to shop from anywhere and at any time (Alba et al., 1997). Furthermore, consumers may browse a multitude of products without having to visit a physical store, as a result, the rapidly expanding e-commerce cyberspace offers firms and customers a more efficient buying channel. In this situation, consumers are more interested in how social media may improve their shopping experiences to help them make better purchasing decisions (O’Cass and Fenech, 2003). When consumers shop online, excessive or unnecessary purchases may develop compulsive buying (Kukar-Kinney et al., 2009). Compulsive buyers prefer to use the Internet or social media to avoid others discovering their dysfunctional purchase behavior and to avoid being exposed to others’ opinions; as a result, compulsive buyers who usually shop to improve their feelings or relieve psychological pressure are more prone to Internet compulsive buying intentions and behaviors (LaRose, 2001).

Because of the wide variety of products available online and the speed to complete purchase, a rising number of consumers are turning to online shopping; nonetheless, online shopping can become addictive. According to LaRose and Eastin (2002), several types of deviant consumer behavior have evolved from the electronic-commerce environment as a result of the influence of mass media and the change in sorts of buying activities. Greenfield (1999) has stated that the percentage of compulsive buyers is larger in the social media-shopping environment than in traditional (offline) retailers because compulsive customers seek a private opportunity to shop without the influence of others. The Internet’s concealment facilitates the compulsive buyer’s avoidance of social contact and delivers quick gratification; nevertheless, as is typical of compulsive buyers, they may experience feelings of loss and worry shortly after purchase. Users publicly show their lifestyle and belongings to their connections on social media (Chu, 2011) pushing users with lower self-esteem and fragmented identities to be more involved in compulsive shopping to demonstrate their status to others and to resolve their inner insecurities.

### Conspicuous Buying Behavior in Relation to Social Media

Buying luxury products can be a case of conspicuous consumption and a first theory on this was framed and developed by Veblen (1899). Conspicuous buying is defined as the act of showing off expensive and luxury items or services (Sundie et al., 2011) motivated by social factors such as impressing others, improving one’s social status and gathering prestige through objects rather than quality features (Nunes et al., 2011). Scholars have frequently employed social comparison theory (SCT) as a theoretical lens through which to analyze conspicuous and impulse buying within social media (Liu et al., 2019). Users frequently engage in comparisons between themselves and influencers who are often “idealized” version of their self (Richins and Rudmin, 1994). Because of the level of control, the user has over the information that is posted, social media has a direct impact on conspicuous consumption. Conspicuous consumption on social media leads to enhanced levels of self-esteem because people are more likely to share only positive information about themselves (Gonzales and Hancock, 2011), which is frequently received with favorable comments (Ellison et al., 2007). These favorable social effects are likely to diminish an individual’s self-control and, as a result, contribute to a preference for hedonic or opulent products or experiences over utilitarian ones as a result of social media use (Khan and Dhar, 2006).

Other research found a link between improved self-esteem and conspicuous buying decision making (Wilcox et al., 2011; Thoumrungroje, 2018) agrees that because social media facilitates interaction between people who have deep links, it also facilitates exposure to direct and indirect product recommendations from reliable sources, boosting the temptation to make impulsive rather than required purchases. This discovery is in line with the findings of another study, which showed that using social media boosts self-esteem while also stimulating the desire to spend (Wilcox and Stephen, 2013). Increased social media engagement (also known as “social media intensity”), increases an individual’s consumption of conspicuous products. The more time someone spends on social media, the more likely they are to make indulgent purchases. In conclusion, research shows that social media increases the frequency of conspicuous consumption in a variety of ways. To begin with, having more control over the information that is given and receiving good feedback leads to greater self-esteem, which in turn encourages conspicuous purchasing. Second, buying motivation is raised as a result of interactions between people with strong links and increased exposure to product recommendations through electronic word-of-mouth.

### Impulse Buying Behavior in Relation to Social Media

Impulse buying is a consumption behavior that results from an immediate response to a stimulus with no consideration of potential purchase outcomes (Rook, 1987). The negative consequences of such unplanned shopping include debt, stress and anxiety (Podoshen and Andrzejewski, 2012). Impulse buying typically occurs when a person’s desire competes with his or her super-ego which seeks to overcome the short-term impulse to purchase goods without purposeful thought (Podoshen and Andrzejewski, 2012).

According to the literature in this field, social media have a strong influence on behaviors such as impulse buying (Aragoncillo and Orus, 2018). Among the content regularly shared by social media users are evaluations of the goods and services they consume (Sharma et al., 2010). This behavior often involves users sharing pictures of their transactions and offering suggestions, which incentivize unplanned and impulse purchases (Xiang et al., 2016). These posts are not limited to influencing buying behaviors, however, but also help to create favorable brand images which in turn can stimulate impulse buying (Kim and Johnson, 2016).

#### Conceptual Framework

As outlined by Ducoffe (1996), SCM provides users with updated information, and users’ perceptions revolve around the usefulness and informativeness of the content. Previous studies reviewed in this research have indicated that individuals with high levels of materialism tend to be heavy users of social media and more positively open to SCM, and that users with high social media engagement and that have more positive attitudes toward SCM may be more likely to develop negative consumption behaviors. The hypotheses tested in this study are drawn from the conceptual framework below:

#### Hypotheses

Materialists compare themselves with those who appear to have more money than them and place great stock in what these people say and display, creating feelings of inequality, injustice, frustration or envy (Sirgy, 1998). Yoon (1995) identified a positive correlation between materialism and general attitudes toward brands advertisements, suggesting the theory that more materialistic people may have more positive attitudes toward advertising. The present study supports Yoon’s views on the positive relationship between materialism and positive attitudes toward SCM, but also embraces the assumptions of Richins and Dawson (1992) and of Wang and Wallendorf (2006) that materialism is a permanent personal value that is likely to affect attitudes such as advertising attitude in general. Therefore, this study expects the following relationships:

*H1: Facebook and Instagram users with higher materialistic values will have more positive attitudes toward social media content (SMC).*

Materialistic individuals make more social comparisons between their own and others’ standards of living. These comparisons in turn drive them to increased social media usage, since social media provides users with access to the information of friends and acquaintances (Haferkamp and Krämer, 2011). This study, therefore, hypothesizes that:

*H2: Facebook and Instagram users with high materialistic values will have higher scores for social media intensity usage behaviors (SMI).*

According to the social comparison theory (Festinger, 1954), individuals have a fundamental desire to determine their own beliefs and abilities by comparing themselves to others. There can be upward or downward comparisons. The upward comparisons with “better” others lead to negative self-assessments, while downward comparisons with “worse” others lead to self-enhancement and more self-confidence. Since advertising content is mostly filled with idealized images, exposure to advertising messages may result in negative comparisons and in an increased need to acquire the advertised products or services (Lee et al., 2016). As a result of social comparison through sponsored content, brand posts and users’ reviews, users will have a negative self-evaluation and may also end up more likely to buy advertised products. Therefore, this study hypothesizes that:

*H3a: Facebook and Instagram users with more positive attitudes toward social media content*

*(SMC) will have higher scores for compulsive buying.*

In his research, Trigg (2001) stated that a significant factor influencing conspicuous consumption was a form of individual emulation of a social group perceived to be in a higher place in the social hierarchy. Facebook and later Instagram usage has boomed partly because both platforms allow their users to present attentively crafted presentations of their idealized selves to others (Ellison et al., 2006). The idealized self is linked to the possessions and the experiences showcased in the users’ profiles. Conspicuous consumption provides a symbolic representation of a prestigious position within the social network, providing a psychological advantage to individuals during the consumption process. Many consumers purchase goods because of the symbolic meanings they attach to those goods (Tutgun-Ünal, 2020). This study therefore hypothesizes that:

*H3b: Facebook and Instagram users with positive attitudes toward social media content (SMC) will tend to display more conspicuous buying behavior.*

According to LaRose (2001), the online environment stimulates impulse buying. E-commerce can undermine customer purchase restraints through its high engagement features with enhanced product stimuli, loyalty programs and chat rooms, steering customers toward impulse purchases. The perception of watching customized social media ads, created by studying customers’ online profiles and habits and then matching ad content to the users’ identified preferences, interests, or history of browsing, is a factor affecting impulse buying behaviors. Therefore, as many studies have revealed, when consumers are highly engaged with a media vehicle, they can be more responsive to brand advertisements (Wang and Wallendorf, 2006). This study therefore hypothesizes that:

*H3c: Facebook and Instagram users with more positive attitudes toward social media content (SMC)* will have higher scores for impulsive buying.

In an effort to resolve identity fragmentation caused by the images seen on social media, heavy social media users who experience anxiety and lower self-esteem may resort to more compulsive buying behavior (Tutgun-Ünal, 2020). SMI was found to be positively related to online compulsive buying behavior and financial anxiety. Results showed that online compulsive buying mediated the positive relationship between excessive use of social media and financial anxiety (She et al., 2021). Therefore, this study hypothesizes that:

*H4a: Facebook and Instagram users with high social media intensity usage behavior (smi) scores will have higher scores for compulsive buying*

Khan and Dhar (2006) found that being an heavy users of social networking sites can enhance the users’ self-esteem while also influencing their self-control. The enhancement in the self-esteem may result in an individual’s choice of placing themselves higher in the social hierarchy through the purchase of luxury goods online, thereby giving rise to consumers’ indulgent behavior. This study therefore hypothesizes that:

*H4b: Facebook and Instagram users with high social media intensity usage behavior (SMI) scores will tend to display more conspicuous buying behavior.*

The findings of Khuong and Tran (2015) showed that consumers’ social media usage intensity becomes a strong factor behind indulging in impulse purchases. Dodoo and Wu (2019) developed a conceptual model to determine the predictive power of social media on impulse buying tendency and found a positive influence. This study therefore hypothesizes that:

*H4c: Facebook and Instagram users with high social media intensity usage behavior (SMI) scores will have higher scores in impulse buying.*

Attitudes toward SCM are often related to the concept of luxury and exclusiveness. The content that represent high quality and prestige positively attract materialistic individuals (Czarnecka and Schivinski, 2019). This is particularly true of advertisements that receive significant investment from advertisers and where endorsers are celebrities who often display expensive goods and exclusive lifestyle experiences. These ostentatious displays are often envied by materialistic people who place high value and meaning on material possessions and exclusive things. This study therefore hypothesizes that:

*HM1: the relationships between materialism and the three negative consumption behaviors will be positively mediated by the roles of attitudes toward social media content.*

Engaged social media users compare themselves to other users. As a result, they spend more time and contribute more in terms of content creation. Users who invest more time and are more interested are more likely to be exposed to texts, comments, tweets, and reviews from experts, influencers, and other users. This exposure can then push them to gain more social proof, believing that the greater the number of people who find an idea or a product interesting, the more likely it is to be true. Furthermore, heavily engaged social media users tend to make purchases that are more driven by emotions and by what other users are doing or showing rather than making a rational purchase decision. This study therefore hypothesizes that:

*HM2: the relationships between materialism and the three negative consumption behaviors will be positively mediated by the roles of social media intensity, which will be stronger than the mediating effect of attitudes.*

Materialism is one determinant factor in compulsive buying (Yurchisin and Johnson, 2004). It influences the buying decisions of consumers. Materialism is among the main causes giving rise to compulsive buying tendencies (Dittmar, 2005). This study therefore hypothesizes that:

*H5a: Facebook and Instagram users with high materialistic scores will have higher scores for compulsive buying.*

Suresh and Biswas (2020) discovered that social media addiction and emotions like loneliness, depression, low self-esteem and anxiety encouraged users to go ahead and maintain relationships in a virtual space rather than engage in face-to-face interactions. Social media addiction can also be positively related to online compulsive Buying. O’Cass and Frost (2002) found that customers with stronger materialistic impulses were using clothing to control impressions, placing trendy clothing in a central role in their lives and using it to communicate their success to others. Furthermore, the research findings of Arndt et al. (2004) showed that the acquisition and display of status-oriented materials play a role in Western social structures, thereby reinforcing the links between buying behavior and society. Consumers may be led to believe that the possession of certain conspicuous goods is important for their upward social mobility. Therefore, this study hypothesizes that:

*H5b: Facebook and Instagram users with high materialistic values will tend to display more conspicuous buying behavior.*

Research findings reveal that psychological motivations such as envy, materialism, narcissism, and social comparison are of significant influence in enhancing users’ online conspicuous consumption (Qattan and Al Khasawneh, 2020). Troisi et al. (2006) showed that highly materialistic people have higher impulse buying tendencies. In addition, they argued that materialistic people buy goods because of the positive sensation it provides. Different working lines indicate that materialism is a technique to minimize adverse effects of unmet psychological needs by enhancing self-esteem (Dittmar et al., 1996). Therefore, this study hypothesizes that:

*H5c: Facebook and Instagram users with high materialistic values will* Have Higher Scores *in* Impulse Buying.

## Materials and Methods

**FIGURE 1:**
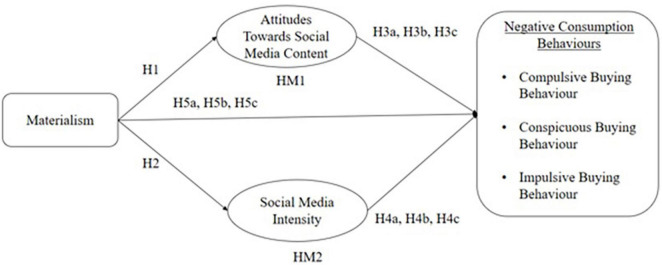
Conceptual framework.

**FIGURE 2:**
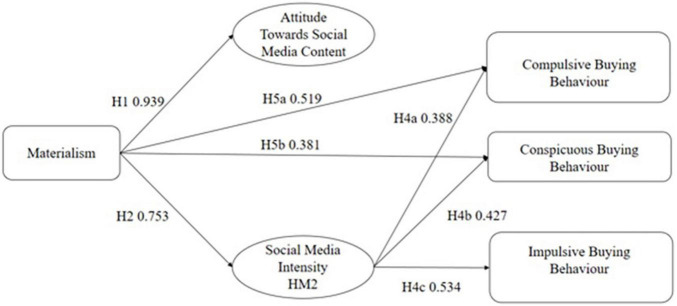
Conceptual framework significant effects.

This study adopts a deductive quantitative method to test the impacts of social media users’ levels of materialism, attitudes toward advertising and social media usage intensity on negative consumption behaviors (Moran and Gossieaux, 2010). The proposed framework (Figure 1) aims to measure the paths of mediation between materialism and three negative consumption habits. While in Figure 2 you may observe all the significant paths within the conceptual framework. This study adopted a correlational cross-sectional, questionnaire-based research design to study the mediation role of attitude and SMI in the relationship between materialism and three negative consumption behaviors. Using Facebook and Instagram a survey was conducted in June 2021 to collect data from Thai social media users commenting on both Facebook and Instagram pages of the three most well-known Thai e-commerce platforms. The online questionnaire consisted of two sections for data collection through a convenience sampling technique. In the first section, participants were asked to report their age, gender and other demographic values, they were also asked to report how much time they spend on social networking sites daily. Additionally, there were two self-reported screening question were asked to ensure the participant fulfilled the purpose of this study (“indicate whether you actively use social media” and “indicate whether you actively engage with content on social media”), those who answered “yes” for both questions were included in the study and proceed to the second section of the questionnaire. The second section consisted of items that measured respondents’ levels of materialism, attitude toward SCM, SMI, compulsive, conspicuous, and impulse buying behavior. The questionnaire was translated from English to Thai using forward-backwards method.

### Data Sampling

As this study is one of the few focusing on harmful consumption behaviors in the social media domain, the interacting variables were restricted to a single country context to eliminate the macro environmental diversity that exists among countries (Yang and Ganahl, 2007). The rationale for selecting Thailand as the study setting was that it has one of the world’s highest levels of social media engagement and social media penetration (Katchapakirin et al., 2018). The standard and sophisticated methods of statistical analysis, including structural equation modeling, recommend a sample size of 200 as a fair number and 300 as a good number of respondents (Tabachnick and Fidell, 1996). The authors also used at least 10 respondents for each item contained in the measurement’s scales. A number of related social media studies (Ahmed et al., 2019) used a sample size of less than 400 and close to 300 with a response rate of 14.59–22%. These studies also used structural equation modeling as a tool of analysis. The sample size was sufficient to meet the target statistical power of 0.8 according to the results of the power analysis. *A priori* estimation to determine sample size was also employed. First, during the research design stage, *a priori* sample size estimation was used to determine the minimal sample size required to avoid type I or type II errors (e.g., Beck, 2013). Second, the minimal sample size was calculated using six latent variables with a probability level of less than 0.05, a power level of 0.8, and a 0.15 effect size (Cohen, 2013). Previous research has used a sample size similar to the one used in this study (Zheng and Lee, 2016; Sharif and Khanekharab, 2017; Hasan et al., 2018; Sharif et al., 2019). The response rate for any social science study in Thailand is relatively low, and 20% could be optimistic. Therefore, this study attempted to yield approximately 400 respondents which would be more than sufficient to satisfy the statistical recommendation for the proposed testing tools and analysis.

The individuals in the sample had to meet the criteria of being Thais who use social media as their primary source of data. The sample frame was developed by using Audience Insights, a tool designed to help marketers learn more about their target audiences, including aggregate information about geography, demographics, purchase behavior, and other relevant criteria. Facebook and Instagram were selected as the two social media platforms from which this study’s sample would be sourced as they are among the top 5 most-used platforms in Thailand for percentage of monthly active users. For the data sample source, this study was based on the e-commerce platforms that are currently popular in Thailand and from which users tend to be more likely to purchase products. Higher numbers of likes in a Facebook page or followers in an Instagram page were the factors used for identifying the larger and more influential e-commerce platforms (Arora et al., 2019). For this study, the author chose Lazada, Shopee, and Chillindo, which offer products from numerous brands. For the brand-generated content construct, we use the messages posted by the e-commerce platforms and not the brands sold on their platforms to minimize the effects of brand-specific factors (Townsend et al., 2013) (e.g., brand reputation, their own social media activities, brand-influencers), which can potentially bias the interpretation of the results. Convenience quota sampling was used in this study as three age- and two gender-based subgroups of users were encountered from the overall Thai social media user population of 55 million users (Digital, 2021). The three age categories were 18–34 years (56.5%), 35–54 years (28.8%), and over 55 years (14.7%) with a total gender count of 49.3% males and 50.8% females.

### Questionnaire Development and Construct Measures

The reliability and validity of the measures proposed have been assessed and their Cronbach’s alpha scores are all above the 0.70 threshold (Nunnally, 1975). Content validity involves the subjective assessment of the measurement scales or items included in the variables (Malhotra, 1981). The content domain was well defined and reflected in each of the construct measures (Sirgy, 1999). For this study, almost all of the construct measures were derived from scales used in published studies with a Cronbach’s alpha reliability of no less than 0.70. The content validity of the questionnaire was also overseen by a panel of three academic scholars. For measuring each theoretical construct in the proposed model, the measurement variables are briefly discussed below.

Materialism is conceptualized as an innate personality trait based on Belk’s three measures of materialistic traits: possessiveness, non-generosity, and envy. As the operational definition specifies, the items were extracted from Belk (1985) research where alpha reliability was reported as 0.73. The scale is appropriate to the study as it defines materialism as an endogenous personality trait of individuals which is the main conceptualization adopted in this study. The four points Likert scale showed high construct reliability and was tested in a dimension similar to that of the study (Chu et al., 2016).

Attitudes toward SCM is defined as “a learned predisposition to respond in a consistently favorable or unfavorable manner toward SCM in general” (MacKenzie and Lutz, 1989, p. 49). Attitudes toward SCM can therefore be defined as a consumer’s like or dislike of SCM. The items tested on a 4 points Likert scale used in this study’s survey were extracted from the study of Ling et al. (2010) of which had high Cronbach’s alpha scores (0.93).

Social media intensity captures a user’s level of engagement with social media, not by measuring the amount of time the user spends on the platform or the number of followers the user has but by measuring his or her level of contribution on the platform. As this measure aims to understand the frequency with which users make certain actions while on social media, this study uses a 4-point Likert scale, ranging from 1 Not at All to 4 Very Often. The items used in the survey were extracted from the studies by Li et al. (2016) and Schivinski and Dabrowski (2016) in which alpha reliability was 0.86 and 0.91, respectively.

Compulsive buying behavior refers to the making of continued and repeated purchases characterized by excessive preoccupation with or poor impulse control over shopping, leading to severe adverse social and economic consequences. The items used a four points Likert scale and were extracted from the study by Faber and O’Guinn (1989) in which alpha reliability was 0.87 and it is currently the most well-known and widely used scale on the subject.

Conspicuous consumption behavior is the purchase of goods or services for the specific purpose of displaying one’s wealth. The items used a four points Likert scale and for this measurement were extracted from the work of Eastman et al. (1999) in which alpha reliability was 0.86.

Impulse buying behavior is an unplanned decision made right before making a purchase. Price discounts are powerful triggers of impulse buying. The items for this measurement used a four points Likert scale and were extracted from the study by Rook and Fisher (1995) in which alpha reliability was 0.88. This scale has been recognized as highly reliable in the literature on impulse buying behavior (Mandolfo and Lamberti, 2021).

### Data Collection

This study’s questionnaire was assessed by three experts, all of whom are management- level faculty members who are active in survey research. This helped assure the scale’s validity within the context of this study. A small pilot test was conducted with 50 respondents to assess the statistical reliability of the measures for the concepts. The survey was then submitted to social media users who met the eligibility criteria of being Thai, regular Facebook and Instagram users, and actively following and interacting with the posts of the three e-commerce platforms on their social media pages. The rationale for selecting the three e- commerce platforms is that they use heavily customized advertisement messages known as retargeting which are thought to have a strong influence on online consumption.

The widely used online survey method was utilized in this study for its ability to collect data efficiently, at minimal expense, and in a short period of time from physically scattered individuals. Distributing the self-administered survey through Facebook and Instagram was deemed the most appropriate data collection method for a widespread research program in the domain of social media (Zikmund, 2000). Every fifth user who had posted a like or reaction under the posts of the selected Facebook pages was selected and every seventh Instagram follower from the selected Instagram pages was drawn systematically for initial contact. If any of the users identified were non-Thai, the researcher would select the next Thai user available. Finally, 3,000 invitations to the survey were sent to the selected participants and a further follow-up message yielded 176 complete responses (85 Males and 91 Females) within 4 weeks of the surveys being sent out. A second message and further follow-up produced an additional 235 (121 Males and 114 Females) responses. Initial responses total 411 (a 14% response rate). Four responses were found to be unusable during the data screening and refinement phase, and they were eliminated. The outlier cases were identified using the remaining 407 responses. Data normality was verified during this process, and seven univariate outlier cases were detected using standardized z scores. With those seven outlier cases removed, the available final sample size for confirmatory factor analysis and structural model testing was 400.

### Data Analysis

This study’s construct measures require measurement of scale reliability and validity. Structural equation modeling (SEM) was used for the confirmatory factor analysis with Amos version 26 followed by specification and estimation of the structural model (Lei and Lomax, 2005). SEM is a powerful quantitative data analytical technique which estimates and tests theoretical relationships between/among latent and/or observed variables and also combines regression and factor analysis (Tabachnick and Fidell, 1996). SEM is also a path analysis method for handling multiple relationships and assessing relationships from exploratory analysis and confirmatory analysis (Hair et al., 1998). It has been used in similar studies for examining the impacts of the predictors of materialism and overconsumption (Alzubaidi et al., 2021). For this study the researchers used SPSS Version 26 and Amos for structural equation modeling.

## Results

A non-probability sampling technique with convenience sampling method was used for data collection (Malhotra, 2006) in which online questionnaires were sent to social media users interacting on the well-known social media profiles of e-commerce platforms namely: Lazada, Shopee, and Chilindo. These are three of the most popular e-commerce platforms in Thailand followed on both Facebook and Instagram by a high number of people (Pongpaew et al., 2017). Although demographic data have no bearing on the study’s level of research, this reporting may provide a broad picture of social media users’ involvement in purchasing products or services online. The survey respondents were comprised of 49.3% males and 50.8% females. The age groups of the respondents were 18–34 years (58%), 35–years (28%) and over 55 years (14%). This gender and age breakdown is in line with the relevant proportions of the total number of social media users in Thailand, as indicated in research conducted by Digital (2021). For more information regarding the study demographics (see Table 1).

**TABLE 1:** Profile of the respondents.

Characteristics	Rank	Frequency	Percentage
Gender	Male	197	50.8
	Female	203	49.3
Age	18–34	51	58.0
	35–54	49	28.0
	55 +	51	14.0
Education level	High school	49	18.0
	Some college	51	12.0
	Bachelor degree	49	36.0
	Master degree	51	30.0
	Doctorate	49	4.0
Employment status	Full time	51	16.0
	Part time	49	36.0
	Self employed	51	18.0
	Student	49	28.0
	Unemployed	51	2.0
Device used	Computers	49	6.0
	Smartphones	51	78.0
	Tablets	49	16.0

### Measurements Model

Cronbach’s alphas and composite reliabilities of all measures are above 0.7 (Table 2), suggesting good reliability and internal consistency of all measures (Nunnally, 1978). All average variance extracted (AVEs) are greater than 0.5, and factor loadings are above 0.7, indicating adequate convergent validity of the constructs (Hair et al., 2011).

**TABLE 2:** Evaluations of measures.

Constructs and items quality criteria	Item mean	*SD*	Standardized factor loadings
**Materialism** (Belk, 1985) CA = 0.98, CR = 0.95, AVE = 0.94			
Renting or leasing things is not more appealing to me than owning	2.56	0.902	0.855
I tend to hang on to things I should probably throw out	2.57	0.918	0.844
I get very upset if something is stolen from me, even if it has little monetary value	2.59	0.859	0.843
I get particularly upset when I lose things	2.61	0.901	0.842
I usually lock things up	2.58	0.895	0.838
**Attitudes toward social media content** (Ling et al., 2010) CA = 0.91, CR = 0.95, AVE = 0.74			
Advertising on social media is trustworthy	2.81	1.056	0.884
Advertising on social media is funny	2.83	1.01	0.875
Brand pages on social media play an important role in my buying decisions	2.78	1.035	0.876
I consider users’ content on social media good as it allows me to discover the best deals	2.75	0.964	0.949
**Social media intensity** (Schivinski et al., 2016) CA = 0.91, CR = 0.93, AVE = 0.81			
I comment on text only posts made by brands on social media	2.69	0.935	0.874
I write reviews on brand pages on social media	2.78	0.917	0.841
I click like on pictures posted by other users on social media	2.77	0.924	0.854
I share content posted by other users (Not friends) on social media	2.78	0.898	0.806
I write posts	2.71	0.963	0.927
I update my personal profile (change image/contact information/privacy setting)	2.69	0.933	0.870
I buy products or services directly on social media	2.79	0.881	0.776
**Compulsive buying behavior** (Faber and O’Guinn, 1989) CA = 0.90, CR = 0.93, AVE = 0.95			
If I have any money left at the end of the pay period, I just have to spend it	2.79	0.939	0.816
I often feel others would be horrified if they knew my spending habits	2.77	0.976	0.805
I often buy things online even though I can’t afford them	2.83	1	0.796
I take on debts even if I know I do not have enough money in my bank to cover	2.8	1.054	0.794
I often buy things online in order to feel better	2.77	1.01	0.785
I feel nervous on days I do not shopping	2.74	1.014	0.783
I make only minimum payments with my credit card	2.82	1.043	0.779
**Conspicuous buying behavior** (Eastman et al., 1999) CA = 0.87, CR = 0.88, AVE = 0.89			
I would buy a product online just because it has status	2.84	1.024	0.830
In social media, I am interested in products with status	2.81	1.038	0.830
I would pay more for products if they had status	2.89	1.01	0.821
The status of a product is irrelevant to me	2.74	0.996	0.812
A product is more valuable to me if it has some snob appeal	2.79	1.057	0.792
**Impulse buying behavior** (Rook and Fisher, 1995) CA = 0.83, CR = 0.85, AVE = 0.93			
I make unplanned purchases online	2.73	0.936	0.792
When I see something that interests me on social media, I buy it without considering the consequences	2.75	0.959	0.839
It is fun to buy spontaneously	2.7	0.917	0.828
I avoid buying things I have not planned to buy	2.73	0.957	0.825

Fornell-Larcker criterion evaluation was used to evaluate the discriminant validity of each construct (Table 3). All square roots of AVEs of constructs are greater than the correlations between them and any other constructs, supporting the discriminant validity of all constructs in the model (Fornell and Larcker, 1981).

**TABLE 3:** Fornell-Larcker criterion assessment.

	Materialism	Social media intensity	Attitudes toward social media content	Impulse buying behavior	Conspicuous Buying Behavior	Compulsive buying behavior
Materialism	**0.96**					
Social media intensity	0.753	**0.9**				
Attitudes toward social media content	0.939	0.707	**0.89**			
Impulse buying behavior	0.609	0.694	0.597	**0.96**		
Conspicuous buying behavior	0.845	0.826	0.859	0.632	**0.94**	
Compulsive buying behavior	0.863	0.823	0.881	0.635	0.844	**0.97**

*The bold numbers correspond to the squared roots of AVE which should be higher than any of the correlation with the same latent variable.*

### Results of the Structural Equation Model

All variance inflation factors (VIFs) ranged from 1.365 to 2.207, well below the cut—off point value of 5 (Hair et al., 2011), indicating that collinearity was not a problem in this model. Bootstrapping was performed with 5,000 samples to examine the significance of path coefficients (Table 4). The results in Table 5 show that attitudes toward SCM did not mediate the relationship between materialism and each of the three negative consumption behavior, while SMI was found to positively mediate the relationship between materialism and negative consumption behavior. SMI was the only construct able to positively mediate the relationship between materialism and all three buying behaviors.

**TABLE 4:** Total, direct and indirect effect.

	Materialism		Social media intensity					Attitudes toward social media content	
			
	Total	Direct	Indirect	Total	Direct	Indirect	Total	Direct	Indirect
Social media intensity	0.753*	0.753*	−	−	−	−	−	−	−
Attitudes toward social media content	0.939*	0.939*	−	−	−	−	−	−	−
Impulse buying behavior	0.631*	0.246	0.385*	0.534*	0.534*	−	−0.01	−0.01	−
Conspicuous buying behavior	0.879*	0.381*	0.498*	0.427*	0.427*	−	0.188	0.188	−
Compulsive buying behavior	0.909*	0.519*	0.390*	0.388*	0.388*	−	0.104	0.104	−

*p < 0.05.

**TABLE 5:** Results of structural equation model analysis.

Hypotheses	Standardized (β)	*P*-value	Results
H1: MAT - > SMC	0.563	0.000	**Supported**
H2: MAT - > SMI	0.833	0.000	**Supported**
H3a: SMC - > COM	−0.005	0.94	Not supported
H3b: SMC - > CON	−0.086	0.325	Not supported
H3c: SMC - > IMP	0.065	0.712	Not supported
H4a: SMI - > COM	0.494	0.000	**Supported**
H4b: SMI - > CON	0.576	0.000	**Supported**
H4c: SMI - > IMP	0.881	0.000	**Supported**
H5a: MAT - > COM	0.567	0.000	**Supported**
H5b: MAT - > CON	0.428	0.000	**Supported**
H5c: MAT - > IMP	0.017	0.927	Not supported
HM1: MAT *SMC*COM, CON, IMP			Not supported
HM2: MAT *SMI* COM, CON, IMP			**Supported**

*Sign used to highlight the mediators in the two relationships. Bold words are used only for the supported hypotheses.

The model fit indices indicate that the relative chi square (v = chi square over degrees of freedom) is 3.179 therefore acceptable as it is lower than 5, chi square is significant, however, as the dataset population examined is higher than 200 that is acceptable. GFI is 0.84 slightly lower than the recommended threshold of 0.90 however, still acceptable. SRMR is 0.06 and RMSEA is 0.07 both lower than the recommended 0.08 therefore acceptable. Lastly CFI (0.93) and TLI (0.92) are both above 0.90 therefore we can conclude that the Structural equation model is satisfactory. In the proposed model, eight of the hypothesized paths were found to be significant. In brief, the results of the proposed model output indicated that users’ levels of materialism and SMI were positive predictors of all three behaviors (supporting H4a, H4b, H5a, H5b, and H5c). However, contrary to this study’s predictions, no support was found for the posited positive impact of attitudes toward SCM on negative consumption.

## Discussion and Implications

### Theoretical Implications

By combining the theoretical approach from all extant theories, a new theoretical model has been tested in this study and the results suggest that SMI is an important antecedent of negative consumption habits (Shanahan et al., 2019) specifically conspicuous buying behavior. The results and analysis from this study further imply that all of the identified antecedents extended the directions of use of marketing theories in a new research setting. Theoretical assertions on the role of attitudes toward SCM in developing negative consumption habits did not work in the expected theoretical manner. However, more robust evidence has shown that even though users may enjoy viewing ads and brand-generated content on social media, distrust of these marketing tools eventually leads to reducing these users’ negative consumption behaviors (Cole et al., 2017).

Social media intensity as a predictor of negative consumption behavior signifies that user engagement and involvement in the content creation process are distinctive stimuli for making purchases (Loureiro et al., 2019). The vast majority of the conceptual arguments for these theoretical viewpoints achieved empirical validation through this study. This study has also revealed that in the social media context, the fact that a user enjoys seeing ads does not automatically lead to enhanced purchase. Materialism has a positive significant impact on attitudes toward SCM and SMI, the positive results indicate that more materialistic users typically have more positive attitudes, toward messages while using social media platforms.

### Implications for Social Media Regulating Bodies

This study has shed light on potential measures that governing bodies may put into place to better safeguard the interests of consumers using social media platforms. As SMI has been shown to have a direct impact on enhancing negative consumption habits, tools that measure and limit the virality of misleading marketing campaigns which target vulnerable audiences should be implemented. Social media providers should be required to inform users regarding the sponsor of the ads they view, especially those which have been forwarded, together with the rationale behind why such content has been proposed to them on order to regulate the engagement these posts may get. Furthermore, notification mechanisms for illegal content generated by users and appeal mechanisms for users who have been banned from using such services should be reinforced to prevent unscrupulous companies further targeting users.

It is also important for policymakers to understand how the algorithms underlying content moderation, content ranking, content targeting, and social influence are crucial to assessing the dissemination and amplification of marketing content. Platforms should be required to provide clear information on the number of times content has been curated, moderated, and ordered by an algorithm employed to identify content or accounts that violate the antitrust rules for user-generated content, advertising content, and advertisement targeting.

## Future Research Avenues and Conclusion

As this research was carried out within the Thai context, which is a setting that has been overlooked in the extant academic research, it provides some insights and possible directions for future research by social science academics. Some of the findings can be considered not only as challenging new evidence but also tentative unless verified in follow-up studies. Therefore, one research avenue is further validation in different country contexts. As this research explores only Thailand’s social media users’ perspectives, conducting a study in any similar country context or any other emerging market could further validate the findings.

Second, the knowledge of social media users’ consumption habits could be made more comprehensive by integrating the perspectives of users from different platforms. In addition to this, longitudinal data might be more authentic for validating the findings.

Third, this study intentionally did not choose to focus on a specific category of products or services as the researcher was more interested in theory testing. However, future studies may further test the framework on specific groups of products or services to develop a more robust understanding of social media users’ consumption behaviors.

This study examined the specific impacts of materialism, attitudes toward SCM and SMI on negative consumption behaviors. The findings should be considered with caution as such an empirical attempt is rare and unique in the present research setting. The research design was based on a correlational research design which prevents claiming causal sequence. The findings rely on respondents’ self-reported cross-sectional data, rather than longitudinal data. This may not reflect changing situations and the series of relationships between users and their consumption behaviors over time. The cross-sectional data may be affected by the respondent’s predispositions regarding any events that have happened in the past or by their mental position at the time of completing the questionnaire.

The data have been collected using a convenience sample. This facilitated data collection and control of diversity, but also limits the generalizability of the findings. The data have been collected only from Thai social media users who were following Lazada, Shopee, and Chilindo pages on Facebook and Instagram. The findings from specific group might not represent the total picture of social media consumption behaviors in all their respects. While acknowledging such limitations, this research exhibits an effective comprehension of negative consumption. Accordingly, the study authenticates the developed framework. This also highlights how social media users should be better protected.

## Data Availability Statement

The raw data supporting the conclusions of this article will be made available by the authors, without undue reservation.

## Ethics Statement

The studies involving human participants were reviewed and approved by the IRB Committee Mahidol University. The patients/participants provided their written informed consent to participate in this study.

## Author Contributions

AP took care of data collection, data analysis, and writing the manuscript. MA provided substantial inputs for improving the statistical analysis of the manuscript. RS took care of editing and proofreading. All authors contributed to the article and approved the submitted version.

## Conflict of Interest

The authors declare that the research was conducted in the absence of any commercial or financial relationships that could be construed as a potential conflict of interest.

## Publisher’s Note

All claims expressed in this article are solely those of the authors and do not necessarily represent those of their affiliated organizations, or those of the publisher, the editors and the reviewers. Any product that may be evaluated in this article, or claim that may be made by its manufacturer, is not guaranteed or endorsed by the publisher.
